# Caries imaging using T1 StarVIBE and PETRA sequences compared to normal dental work-up: a proof-of-concept study

**DOI:** 10.1007/s11282-026-00908-8

**Published:** 2026-02-24

**Authors:** Gintare Burian, Jonas Stief, Tobias Greve, Ulrich Grosse, Adib Al-Haj Husain, Gustav Andreisek, Monika Probst, Egon Burian

**Affiliations:** 1 Swiss Biohealth Clinic, Kreuzlingen, Switzerland; 2 Department of Diagnostic and Interventional Radiology, Cantonal Hospital Frauenfeld, Frauenfeld, Switzerland; 3 Department of Neurosurgery, LMU University Hospital, LMU Munich, Munich, Germany; 4 Clinic of Cranio-Maxillofacial and Oral Surgery, Center of Dental Medicine, University of Zurich, Zurich, Switzerland; 5 Department of Cranio-Maxillofacial and Oral Surgery, University Hospital Zurich, University of Zurich, Zurich, Switzerland; 6 Department of Cranio-Maxillofacial Surgery, GROW School for Oncology and Reproduction, Maastricht University Medical Centre, Maastricht, The Netherlands; 7 University of Zurich, Zurich, Switzerland; 8 Department of Diagnostic and Interventional Neuroradiology, Technical University of Munich, Munich, Germany; 9 Department of Diagnostic and Interventional Radiology, University Hospital of Zurich, Rämistrasse 100, Zürich, 8091 Switzerland

**Keywords:** Magnetic resonance imaging, Caries detection, Dental imaging

## Abstract

**Objectives:**

To compare caries detection using T1-based Free-breathing 3D Stack of Stars GRE (StarVIBE) and T1-weighted pointwise encoding time reduction with radial acquisition (PETRA) MRI sequences with the gold standard consisting of a combination of clinical evaluation and bitewing radiography.

**Materials and methods:**

Included subjects were evaluated clinically by a dentist for the occurrence of caries. All subjects underwent bitewing radiographs and MRI of the viscerocranium, including 3D StarVIBE and 3D PETRA sequences. Caries lesions were evaluated for penetration depth (D1-D4). Signal intensity (SI) was measured in dental hard tissue, the pulp and caries lesions.

**Results:**

80 teeth were analyzed. Among these, 10 showed caries during clinical assessments and bitewing imaging, all of which were also detected on MRI. Furthermore, 5 additional lesions were identified exclusively on MRI. Overall image quality and diagnostic confidence were significantly higher in PETRA than in StarVIBE (4.7 vs. 3.9, p = 0.006). The SI for caries lesions was significantly higher in the PETRA sequence (-0.5 a.u. ± 1.5 a.u. vs. -4.2 a.u. ± 4.0 a.u., p = 0.002). Caries lesion volume was also significantly larger in PETRA (18 mm^3^± 14.8 mm^3^) compared to StarVIBE (11 mm^3^± 14.8 mm^3^, p = 0.003).

**Conclusion:**

MRI identified all caries lesions that were detected both clinically and through bitewing imaging, along with additional lesions exclusive to MRI. The PETRA sequence outperformed StarVIBE in quantitative caries visualization.

## Introduction

Until now the detection of caries relies on the combination of clinical examination and bitewing radiography (BR). While effective, conventional radiography inherently involves ionizing radiation, posing a cumulative risk, particularly for pediatric patients or individuals requiring frequent diagnostic imaging. Furthermore, traditional radiographs may have limitations in detecting early-stage lesions, especially in occlusal fissures or under existing restorations, and provide limited information on soft tissue involvement or pulpal status. The literature on the use of magnetic resonance imaging (MRI) for primary and secondary caries detection remains limited [1–3]. In clinical practice, MRI typically is utilized in severe cases of exacerbation of inflammatory processes affecting soft tissues in the proximity of the alveolar ridge [4]. In recent years, the implementation of MRI has attracted attention across multiple dental specialties, including oral surgery, periodontology, and orthodontics [5–8], demonstrating its versatility and potential for broader applications, including cariology.” There is literature comparing T1-based MRI imaging for the detection of osseous pathologies, which shows promising results, particularly in identifying furcation defects and bone loss associated with periodontitis [8, 9]. Additionally, research has highlighted the potential of water-sensitive MRI sequences to detect subtle changes, such as edema in alveolar bone or the presence of dentin fluid leakage or saliva penetration, which may indicate caries lesions [10–12].

In recent years, advances in dental MRI have opened new opportunities for the early, radiation-free detection of dental caries. Unlike conventional radiography, MRI can visualize not only mineral loss but also changes in water content and soft tissue inflammation, providing a more comprehensive picture of lesion activity and pulpal involvement. This makes MRI particularly valuable in pediatric or repeatedly imaged patients. In pregnant patients, non-contrast MRI may be considered when clinically indicated and when no suitable alternative imaging modality is available, in accordance with current safety recommendations, while strictly avoiding gadolinium-based contrast agents. Consequently, MRI-based caries evaluation may support earlier diagnosis and personalized, minimally invasive treatment planning.

The use of ultra short echo time (UTE) and zero echo time (ZTE) sequences has been described by Weiger et al. and Bracher et al., emphasizing the great potential of MRI in the detection of dental defects associated with caries lesions [1, 2]. These sequences not only provided comparable sensitivity to dental radiographs for visualizing caries but also enabled the detection of occlusal and approximal lesions that are often missed during the standard clinical assessments [1]. However, one of the main drawbacks of clinical implementation was the long protocol time of nearly 20 min (7:50 min 3D UTE), which prompted clinicians to avoid further investigations.

This proof-of-concept study aimed to evaluate the diagnostic potential of two rapid MRI sequences—the 3D T1-weighted StarVIBE and the 3D ultra-short echo time PETRA—for the detection of dental caries. The StarVIBE sequence provides high soft-tissue contrast and robustness against motion artifacts, making it suitable for depicting the pulp-dentin interface, while PETRA achieves exceptional hard-tissue contrast with near-zero echo times, enabling improved visualization of enamel and dentin structures. By comparing both sequences against the conventional gold standard of dental examination with bitewing radiographs, this study sought to explore their feasibility, diagnostic value, and potential role in future dental imaging workflows.

## Materials and methods 

### Patient selection

The study adhered to the STROBE guidelines for observational research and was conducted in compliance with the principles of the Declaration of Helsinki. Written informed consent was obtained from all participants. The prospective analysis was approved by the cantonal review board of Thurgau, Switzerland (Ethikkommission Ostschweiz) (EKOS 24/036).

All subjects were scanned in the course of sequence optimization purposes. This design decision inherently positioned the study as a preliminary investigation to evaluate the technical performance and initial diagnostic potential of the MRI sequences, rather than a definitive comparative trial designed to establish statistical superiority over established methods. Participants were consecutively recruited from volunteers undergoing MRI for sequence optimization purposes who also received routine dental examination including bitewing radiographs. Inclusion criteria were the presence of natural teeth and the availability of both MRI and bitewing radiographs. Exclusion criteria were extensive metallic restorations causing non-diagnostic artifacts.

### Dental examination

Dental examination was performed by one dentist (G.B., 7 years of experience) at a private dental practice, and therapeutic decisions were based on the standard practices of dental care. In 5 cases, with caries lesions only detected in MRI, patients were informed by the dentist about the imaging findings and the potential consequences when not treating the identified lesions. One patient opted to treat 3 lesions based on the MRI findings.

### MRI acquisition

All participants underwent MRI scans using a 3-Tesla scanner (Magnetom Vida; Siemens, Erlangen, Germany) equipped with a specialized 64-channel head-neck and spine coil (BioMatrix; Siemens, Erlangen, Germany). The imaging protocol included a 3D isotropic StarVIBE sequence (acquisition time: 5.21 min, voxel size: 0.6 × 0.6 × 1.1 mm³, recon voxel 0.6 × 0.6 × 0.6 mm³, TE: 2.5 ms, TR: 5.2 ms, acceleration factor: non, 102 slices) and a 3D isotropic PETRA sequence (acquisition time: 5.03 min, voxel size: 0.59 × 0.59 × 0.59 mm³, recon voxel: 0.59 × 0.59 × 0.59 mm³, TR: 5.0 ms, TE: 0.07 ms, acceleration factor: non, gap: 0 mm, Slices 256, water-fat shift: 1503/289 Hz). Images were obtained in the axial plane and subsequently reformatted into sagittal and coronal orientations.

### Bitewing radiographs

Bitewing radiographs (BR) were acquired using a standardized intraoral technique with a digital X-ray system (Heliodent Plus, Dentsply Sirona) in combination with a digital intraoral sensor (Xios XG Supreme, size 2). Rectangular collimation and commercially available bitewing holders were used to ensure reproducible positioning and minimize projection errors. Exposure parameters were set to 60 kV, 7 mA, and an exposure time of 0.08 s, in accordance with the manufacturer’s recommendations. Images were obtained with the sensor positioned parallel to the tooth crowns and the X-ray beam oriented perpendicular to the interproximal spaces.

Radiographs were evaluated on a calibrated diagnostic monitor by a dentist (G.B.) and a radiologist (J.S.) under standardized viewing conditions. Caries was assessed according to established radiographic criteria, focusing on radiolucencies within enamel and dentin at approximal and occlusal surfaces.

### Qualitative image analysis

Visibility of caries lesions, hard tissue-pulpa junction, and tooth-to-soft-tissue contrast was evaluated using a 5-point Likert scale (1 = poor, 2 = below average, 3 = fair, 4 = good, 5 = excellent). Overall diagnostic image quality, image artifacts, and diagnostic confidence for caries detection were graded using the same five-point Likert scale. This evaluation method was applied to assess the effectiveness of both MRI sequences in visualizing carious lesions and the anatomical structures of the teeth.

### Quantitative image analysis

The 3D StarVIBE and PETRA sequences were evaluated for caries lesions. SI was calculated using the following formula:

$$\:[\mathrm{S}\mathrm{I}=\frac{{\mu\:}_{\mathrm{Lesion}}-{\mu\:}_{\mathrm{Muscle}}}{{\sigma\:}_{\mathrm{Lesion}}}]$$

Where:

- $\:{\mu\:}_{\mathrm{Lesion}}$ = Mean Signal Intensity of the Lesion.

- $\:{\mu\:}_{\mathrm{Muscle}}$= Mean Signal Intensity of the Muscle (Masseter).

- $\:{\sigma\:}_{\mathrm{Lesion}}$ = Standard Deviation of the Signal Intensity of the Lesion.

Regions of interest (ROIs) for caries lesions were manually drawn along the lesion borders on axial, sagittal, and coronal reformations, ensuring inclusion of the entire lesion volume. For muscle signal, a circular ROI of approximately 10 mm² was placed within the adjacent masseter muscle, avoiding visible vessels or fat. The standard deviation for noise was derived from the signal intensity within a homogeneous region of the lesion itself, as per standard practice for SI calculation in lesion assessment.

All image analyses, including MRI and conventional X-ray-based radiographs, were conducted by a radiologist with 9 years of experience (J.S.) and a dentist (G.B.) with 7 years of experience. The images were analyzed independently and blinded to other diagnostic information. In cases of significant artifacts, such as those from metallic restorations or motion, the affected teeth were excluded from the analysis. Analyses and segmentations were performed using a certified PACS workstation (IDS7 21.2; Sectra, Linköping, Sweden). MRI images were reviewed at least 8 weeks apart from clinical examination and BR to ensure independent evaluations.

### Statistics

All statistical analyses were conducted using IBM SPSS Statistics software (version 29.0, IBM Chicago, IL, USA). Descriptive statistics, including means, standard deviations, minimum and maximum values, and percentiles, were calculated to summarize patient demographics and key imaging parameters. The normality of continuous variables was assessed using the Kolmogorov-Smirnov test, with p-values < 0.05 indicating deviations from a normal distribution.

For paired comparisons between imaging modalities and Likert scales, the Wilcoxon signed-rank test was applied due to non-normal distribution of most variables. A significance threshold of *p* < 0.05 was used for all tests. Data are presented as mean ± standard deviation unless otherwise stated.

Additionally, inter-observer and intra-observer agreement were analyzed using weighted Cohen’s kappa (κ), with interpretation as follows: poor, < 0; slight, 0–0.2; fair, 0.21–0.4; moderate, 0.41–0.6; substantial, 0.61–0.8; and almost perfect, 0.81–1. Measurements were repeated after 6 weeks.

## Results

### Caries detection, localization and classification

**Fig. 1:**
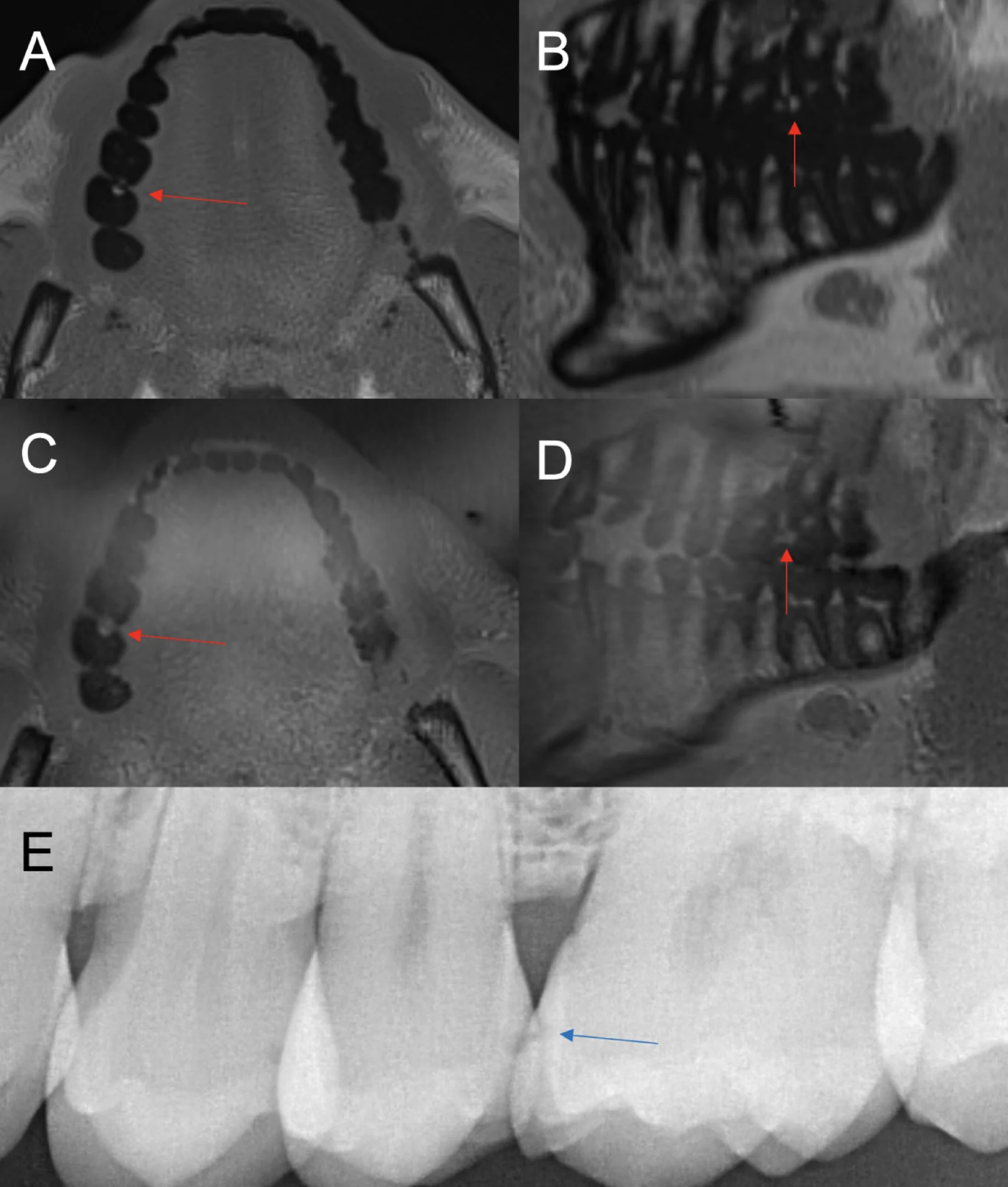
**A** and **B** show the StarVIBE sequence in a patient with caries lesions located approximal in the teeth 16 and 17 in patient 1. The PETRA sequence reveals better visibility of the caries lesions in (**C**) and (**D**). **E** The corresponding radiograph shows an irregularity of the enamel matrix suggestive of caries. Red arrows show the caries in the MRI. Blue arrows show the irregular tooth surface, however superimposition hinders clear diagnosis of caries lesion.

A total of 5 subjects were included in this proof-of-concept study. 15 caries lesions were detected on the MRI scan. For detailed description of cases and number of caries lesions see Table1. 10 of the 15 lesions were identified during a dental examination consisting of inspection by an experienced dentist and BR **(**Fig. 1). The distribution of these lesions was as follows: 2 classified as D1, 4 as D2, and 4 as D3. The remaining 5 lesions, detected only by MRI, were not visible even after the dentist was informed of their exact location. There were no lesions, which could only be identified by dental examination. One of the 5 caries lesions was secondary caries located under a filling, one caries lesion was located in an occlusal fissure, two were in buccal fissures, and one lesion was distal at an upper wisdom tooth.

**Table 1:** Recorded caries lesions and aff ected teeth for the study participants.

Patients	Number of caries lesions	Affected teeth
Patient 1	2	16, 17
Patient 2	1	26
Patient 3	4	14, 26, 36, 37
Patient 4	2	17, 26
Patient 5	6	18, 27, 28, 35, 37, 46

### Qualitative image evaluation

**Fig. 2:**
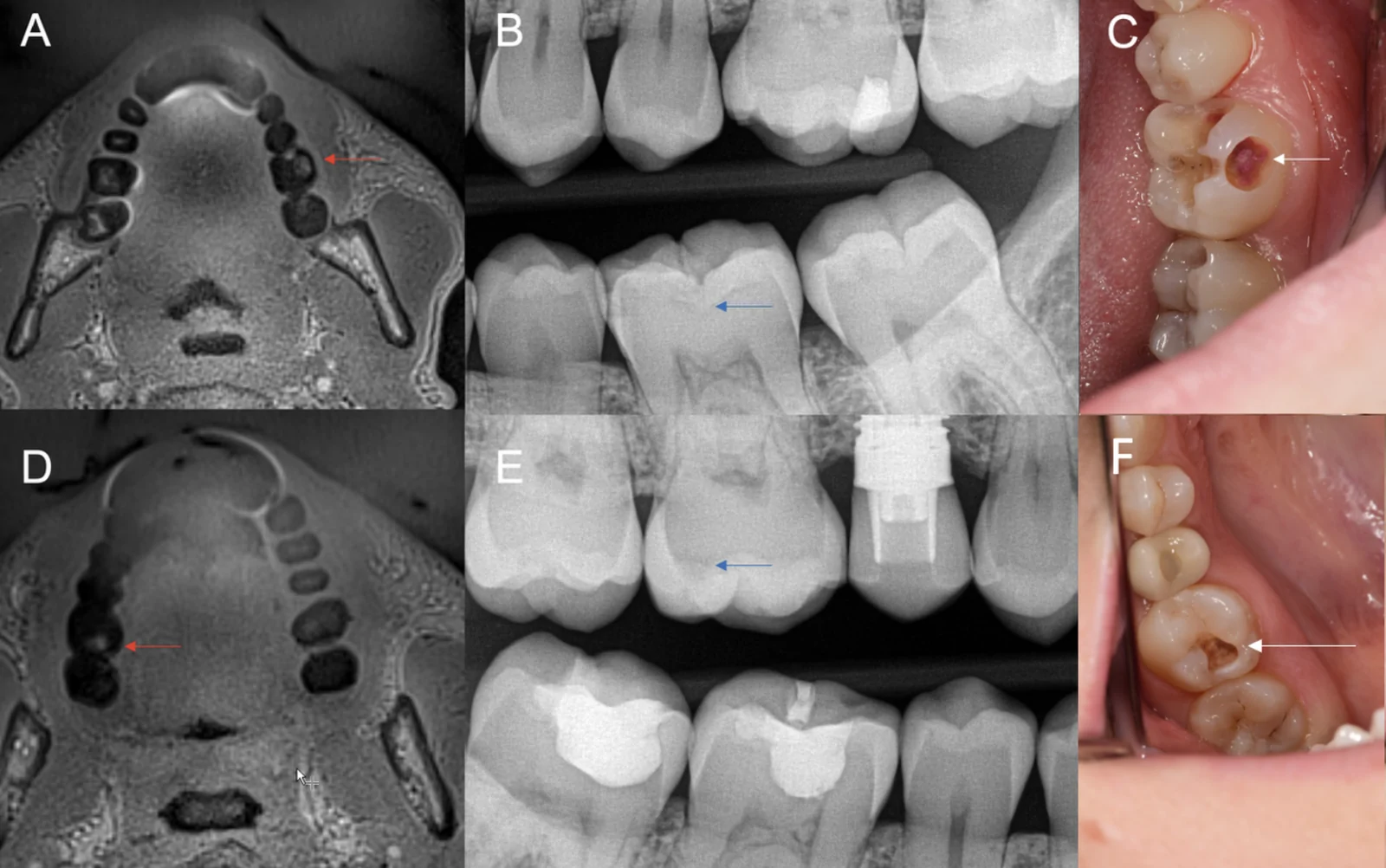
In this figure caries lesions in patient 3 are illustrated. In **A** detection of a large caries lesion on the buccal surface of 36. **B** BR of the second and third quadrant with no clear caries diagnosis. The large caries lesion in 36 is hidden due to superimposition of dental hard tissue. After excavation the true extent of the caries lesion was revealed (**C**). **D** shows an occlusal lesion in 16. **E** the BR of the first and fourth quadrant displays and implant in regio 15 and dental fillings in 46 and 47. The full extent of the caries lesion is shown after excavation (**F**). Red arrows show the caries in the MRI images. Blue arrows show an unclear radiolucency under the enamel layer. White arrows show the true extent of the excavated lesions.

Using the described Likert Scale, PETRA showed significantly better subjective image quality compared to StarVIBE (4.7 vs. 3.9, *p* = 0.006). Based on the MRI findings 3 lesions were excavated by a dentist, revealing the full lesion extent (Fig. 2).

### Quantitative evaluation of caries lesions

Quantitative evaluation of caries lesions revealed distinct differences between the two MRI sequences. Table 2 summarizes the mean values, standard deviations, and statistical significance for lesion volume, lesion SI, dental hard tissue SI, and pulpal SI. A significant difference was found between the PETRA and the StarVIBE sequence with regard to lesion volume (18 mm^3^ ± 14.8 mm^3^ vs. 11 mm^3^± 14.8 mm^3^, *p* = 0.003).

**Table 2:** Quantitative lesion assessment

Parameter	PETRA	StarVIBE	*p*-value
Lesion volume (mm^3^)	18 ± 14.8	11 ± 14.8	0.003
Lesion SI (a.u.)	− 0.5 ± 1.5	− 4.2 ± 4.0	0.002
Dental hard tissue SI (a.u.)	− 13.9 ± 7.0	− 30.4 ± 14.6	< 0.001
Pulp SI (a.u.)	1.3 ± 2.1	− 5.4 ± 5.8	< 0.001

*A.u.* arbitrary units,* PETRA* Pointwise encoding time reduction with radial acquisition, *SI* signal intensity, *StarVIBE* T1-weighted free-breathing 3D Stack of Stars GRE

 In terms of lesion SI, PETRA outperformed StarVIBE with significantly higher values (− 0.5 a.u. ±1.5 a.u. vs. − 4.2 a.u. ±4.0 a.u., *p* = 0.002). In both sequences, dental hard tissue and pulpal cavities were evaluated. Hard tissue displayed significantly higher values in the PETRA sequence compared to StarVIBE (− 13.9 a.u. ±7.0 a.u. vs. − 30.4 a.u. ±14.6 a.u., *p* < 0.001). PETRA also showed a significantly higher SI in the pulp compared to StarVIBE (1.3 a.u. ±2.1 a.u. vs. − 5.4 a.u. ±5.8 a.u., *p* < 0.001).

Additionally, statistically significant differences could be detected between carious lesion SI and dental hard tissue SI for PETRA and StarVIBE sequences (*p* < 0.001). This underlines the high contrast for lesion detection using both sequences.

### Comparison of dental hard tissue and the pulp

In both sequences, dental hard tissue and pulpal cavities were evaluated. Hard tissue displayed significantly higher values in the PETRA sequence compared to StarVIBE (− 13.9 a.u. ± 7.0 a.u. vs. − 30.4 a.u. ± 14.6 a.u., *p* < 0.001). PETRA showed a significantly higher SI in the pulp compared to StarVIBE (1.3 a.u. ± 2.1 a.u. vs. − 5.4 a.u. ± 5.8 a.u., *p* < 0.001). Inter-observer agreement was consistently high in both sequences (κ = 0.8, all *p* < 0.01), intra-observer agreement was even higher (κ = 0.9, all *p* < 0.01),

## Discussion

The present study highlights the potential of 3D T1 StarVIBE and PETRA sequences for caries detection. Our findings suggest that these two sequences can depict caries lesions with promising visualization capabilities that appear comparable to findings from a dental examination in combination with BR for the lesions detected.

Furthermore, both sequences showed lesions that would not typically be detected during a regular dental examination. In this comparison, PETRA demonstrated significantly higher SI for the detection and visualization of caries. Additionally, PETRA was able to detect secondary caries that were missed during clinical evaluation and bitewing imaging. With regard to pulp and dental hard tissue, PETRA again outperformed StarVIBE, showing higher SI. In terms of caries volume, PETRA also showed significantly higher values. However, as the true extent of the lesion cannot be definitively determined, it remains unclear whether either sequence tends to overestimate or underestimate the actual caries volume.

Using T1-based sequences for visualization of osseous or hard tissue pathologies has gained attention in recent years, particularly in musculoskeletal radiology and head and neck imaging [13–15]. In these studies, the potential of MRI was shown for CT-like visualization of fractures and inflammatory changes of the alveolar bone. However, imaging of dental hard tissues using MRI always seemed to be challenging, mirrored in the scarcity of scientific data [1, 2]. A recent study demonstrated the potential of water-sensitive sequences for caries detection when compared to conventional OPT [12]. However, clinical dental assessment combined with BR is the benchmark against which all new imaging modalities must be measured.

The formation of caries lesions is based on the disruption of the protective pellicle and the dental hard tissues in the course of pH drop in the acidic spectrum [16]. In the course of lasting acidic local milieu and steady influx of substrates, an architectural disruption of the enamel matrix is caused [17]. When the caries lesion progresses, the pulp becomes vulnerable to infiltration and infection [18]. The bacterial invasion of the pulp and the occurrence of pulpitis is associated with clinical symptoms like pain and sensation alterations [18]. The clinical evaluation is based on visual and visio-tactile evaluation [19]. The consecutive management of deep caries lesions and associated exposed pulp includes capping techniques and partial pulpotomy or complete pulpectomy with associated devitalization of the corresponding tooth [20].

Besides the clinical gold standard including BR there several experimental attempts like near infrared imaging (NIR) for approximal caries detection predominantly in scientific use [21–23]. The implementation of short MRI protocols for caries detection appears particularly promising, offering significant clinical utility. The diagnostic significance of MRI in caries evaluation lies in its ability to detect both mineral and soft-tissue changes, offering insight into lesion vitality and extension beyond what conventional imaging reveals. This ability to concurrently assess dentin hydration and pulpal edema allows for a more holistic assessment of the carious process. Moreover, MRI can serve as a valuable adjunct in cases where clinical findings and radiographs are inconclusive or discordant, supporting more informed treatment decisions while completely avoiding radiation exposure. For juvenile patients, this radiation-free approach is invaluable, allowing for repeated scans when clinically indicated without cumulative radiation exposure, thereby offering a substantially improved benefit-risk ratio. This is critical for monitoring caries progression or treatment efficacy in a vulnerable population, for example after pulpotomy of primary teeth.

The increasing accessibility of dental MRI scanners in imaging centers, including their use in specific clinical settings, holds great potential for transforming diagnostic paradigms in dentistry. The use of MRI not only shows promise for diagnostic capabilities that may be comparable to the conventional X-ray-based gold standard in certain aspects but also offers distinct advantages considering inflammation extent visualization and early caries detection [12, 24]. Its ability to depict subtle changes in dentin hydration or pulpal edema provides crucial insights into the vitality of the tooth and the true extent of the carious process, facilitating earlier, less invasive interventions and improved patient outcomes.

 [12].

Compared to the studies conducted by Bracher et al., we achieved a reduction in scan time of 50%, with a recent protocol length of 10 min instead of 20 min [1, 3]. For clinical use regarding caries detection, a single-sequence protocol would advantageously reduce scan time to under 5 min while minimizing movement artifacts, particularly in children. Still, at the current point of research MRI should be considered a complementary imaging modality in most cases, generating information inherently concealed in radiation-based cross-sectional imaging and projection radiography [10, 11, 25, 26]. This includes detailed visualization of soft tissues, assessment of pulpal inflammation, and detection of early demineralization changes that may precede visible radiographic signs. This comprehensive diagnostic capability allows clinicians to gain a more holistic understanding of the carious process and its impact on surrounding structures, guiding more precise and timely treatment decisions. Furthermore, the potential for early detection of caries lesions using high-resolution T1-based sequences like UTE and ZTE is substantial, particularly as scan times are further reduced using advanced techniques such as artificial intelligence-based reconstruction, paving the way for wider clinical adoption and integration into routine dental diagnostics.

Despite the highlighted clinical and scientific potential for MRI use in dentistry, further prospective studies are needed to validate the findings of this study. There are methodic limitations of the current study addressed in the following: First, the retrospective nature of our investigation and the small sample size only allow for reporting associative findings and preclude definitive conclusions regarding the statistical superiority or equivalence of MRI over conventional methods. Further, metal artifacts of dental restorations can sometimes reduce image quality and hamper the sensitivity of specific MRI sequences for subtle lesion detection in the proximity of the material. Third, considering the cross-sectional study set-up, the lesion extent and clinical relevance of lesions detected only in MRI cannot be assessed. Fourth, MRI captures hydration-related signal changes and edema formation, which may reflect early demineralization or pulpal edema not visible radiographically. Hence, MRI might overestimate lesion size relative to BR, which only visualizes mineral loss in hard tissue projection. Last, as this was a proof-of-concept study focused on technical feasibility, no multi-reader consensus panel was implemented.

## Conclusion

The results of our study support the clinical implementation of a T1-based PETRA sequence for early caries detection.

## Abbreviations

BR: Bitewing radiographs
NIR: near infrared imaging
MRI: Magnetic resonance imaging
OPT: Conventional panoramic radiography
PACS: Picture archiving and communication system
PETRA: Pointwise Encoding Time reduction with Radial Acquisition
SI: signal intensity
StarVIBE: T1-weighted free-breathing 3D Stack of Stars GRE
UTE: Ultra short echo time
ZTE: Zero echo time
